# Zerumbone, a Phytochemical of Subtropical Ginger, Protects against Hyperglycemia-Induced Retinal Damage in Experimental Diabetic Rats

**DOI:** 10.3390/nu8080449

**Published:** 2016-07-25

**Authors:** Thing-Fong Tzeng, Shorong-Shii Liou, Yu-Cheng Tzeng, I-Min Liu

**Affiliations:** 1 Department of Pharmacy and Master Program, Tajen University, Pingtung County 90741, Taiwan; d850084@yahoo.com.tw (T.-F.T.); ssliou@tajen.edu.tw (S.-S.L.); 2 St. Dominic’s Catholic High School, Kaohsiung City 80288, Taiwan; 2770727@yahoo.com.tw

**Keywords:** diabetic retinopathy, zerumbone, AGEs, NF-κB

## Abstract

Diabetic retinopathy (DR), the most ordinary and specific microvascular complication of diabetes, is a disease of the retina. Zerumbone (ZER) is a monocyclic sesquiterpene compound, and based on reports, it is the predominant bioactive compound from the rhizomes of *Zingiber zerumbet*. The aim of the current study is to evaluate the protective effect of zerumbone against DR in streptozotocin (STZ)-induced diabetic rats. STZ-diabetic rats were treated with ZER (40 mg/kg) once a day orally for 8 weeks. ZER administration significantly (*p* < 0.05) lowered the levels of plasma glucose (32.5% ± 5.7% lower) and glycosylated hemoglobin (29.2% ± 3.4% lower) in STZ-diabetic rats. Retinal histopathological observations indicated that disarrangement and reduction in thickness of retinal layers were reversed in ZER-treated diabetic rats. ZER downregulated both the elevated levels of advanced glycosylated end products (AGEs) and the higher levels of the receptors for AGEs (RAGE) in retinas of diabetic rats. What’s more, ZER significantly (*p* < 0.05) ameliorated diabetes-induced upregulation of tumor necrosis factor-α, interleukin (IL)-1 and IL-6. ZER also attenuated overexpression of vascular endothelial growth factor and intercellular adhesion molecule-1, and suppressed activation of nuclear factor (NF)-κB and apoptosis in the retinas of STZ-diabetic rats. Our results suggest ZER possesses retinal protective effects, which might be associated with the blockade of the AGEs/RAGE/NF-κB pathway and its anti-inflammatory activity.

## 1. Introduction

Diabetes mellitus (DM) has been on the rise, resulting in serious economic, social, and health repercussions. The most common and serious complication of DM is diabetic retinopathy (DR), characterized by vascular alterations including endothelial cells dysfunction, breakdown of the blood-retinal barrier, retinal blood flow changes, ischemia, and neovascularisation [1,2,3]. At early stages, DR has no or mild symptoms. However, if not properly treated, it is very likely that DR progresses to the advanced stage, during which severe pathologies often lead to irreversible blindness [3]. Factors like poor metabolic control and hyperglycemia play an important role in the development of DR since intensive glycemic control generally inhibits the progression of DR [4,5,6]. Unfortunately, many patients have difficulty maintaining the optimal metabolic level. Therefore, even though blood glucose cannot be perfectly controlled, there is a need for the development of new drugs which help modulate the mechanism involved in DR.

The activation of various biochemical pathways result from increased circulating levels of glucose accumulated in the retinal endothelial cells. First, a number of signaling mechanisms have been implicated in the pathogenesis of DR [7,8]. The mechanisms include hyperglycemia-induced inflammatory processes like nuclear factor kappa-B (NF-κB), tumor necrosis factor-α (TNF-α), interleukin (IL)-1β, and IL-6. Second, it is known that vascular endothelial growth factor (VEGF) is a potent proinflammatory and angiogenic factor whose vitreal levels are highly correlated with retinal neovascularization and edema [7,8]. Besides the above-mentioned inflammatory mediators, increases in adhesion molecules—such as vascular cell adhesion molecule-1 (VCAM-1) and intracellular adhesion molecule-1 (ICAM-1)—as well as activation of leukocytes have been found to be related to the progression of DR [7,8]. In fact, in future practice, it can be an appealing treatment option for DR to inhibit the inflammatory pathway [9]. Third, by promoting nonenzymatic glycation and advanced glycosylated end products (AGEs) formation, hyperglycemia is involved in the development of DR [10]. The interaction between receptors for AGEs (RAGE) and AGEs elicits reactive oxygen species generation, the expression of proinflammtory cytokines, and the induction of apoptosis [11,12]. As a result, the inhibition of AGEs formation or the blockade of the AGE-RAGE system is a novel suggested therapeutic target for DR [11,12].

*Zingiber zerumbet* Smith is commonly used as herbal medicine in Asian, Indian, Chinese, and Arabic folklores since ancient times. It a perennial edible ginger with many phytomedical properties [13]. In *Z. zerumbet*, a cyclic sesquiterpene compound zerumbone (1, (2E,6E,10E)-2,6,9,9-tetramethylcycloundeca- 2,6,10-trien-1-one; ZER) can be found abundantly in rhizomes followed by α-humulene and camphene [14]. It is commonly used as a condiment for food flavoring [13]. Several studies revealed that ZER can inhibit the proliferation of colon and breast cancers, with minimal effects on normal cells [15]. ZER has been found to suppress skin tumor initiation and promotion, inhibit inducible nitric oxide synthase and cyclooxygenase-2 expression, suppress free radical generation, and inhibit TNF-α release in activated leukocytes [16]. Through regulation of key genes related to oxidative stress, inflammation, and fibrogenesis, ZER was found to have the ability to mitigate nutritional steatohepatitis [17]. Moreover, the compound has a precious anti-diabetic property that involves antihyperglycemia following inhibition of hyperglycemia-affected proinflammatory factors, chemokines, or adhesion molecules expression in kidney of STZ-diabetic rats [18]. Thus, ZER may be adopted as an adjuvant therapy to control diabetes. That the extract of *Z. zerumbet* is beneficial to amelioration of diabetic retinal damage has been previously explored [19]. However, no scientifically proven data show that ZER is the component of *Z. zerumbet* that has protective effect on diabetic retinal tissues.

The retinal lesions that develop in type 1 diabetes are the same as those in type 2 diabetes while the severity and/or incidence of the lesions may differ [20]. According to observation, streptozotocin (STZ)-induced diabetic rats developed retinal lesions similar to those of humans with diabetes, which has attracted widespread attention to this animal model of human DR [21]. Based on this assumption, the present study was designed to observe the ameliorative activity of ZER on STZ-induced DR in rats and its underlying mechanism.

## 2. Materials and Methods

### 2.1. Isolation and Characterization of ZER

ZER was extracted from *Z. zerumbet* rhizomes by Han-Sheng Pharmtech, Inc. (Pingtung City, Taiwan) under internationally certified Good Manufacturing Practices guidelines. *Z. zerumbet* rhizomes were purchased from a local market in Dongshan, Dongshan Dist. (Tainan City, Taiwan) during September 2014. To confirm the authenticity of the plant material, macroscopic and microscopic examinations, as well as thin-layer chromatography and high-performance liquid chromatography, were adopted. To identify DNA polymorphisms, random amplified polymorphic DNA analysis of *Z. zerumbet* rhizomes supplied was also performed. The voucher specimen (Lot No. 20140923) has been deposited in Han-Sheng Pharmtech, Inc. 

Using the hydrodistillation (steam distillation) method, ZER was isolated according to a method reported earlier [22]. Briefly, fresh *Z. zerumbet* rhizomes were initially cleaned, sliced, later placed in a glass flask containing distilled water, and heated immediately by using the heating mantel. Vaporized steam containing the volatile oil was collected after the flask was connected to special glassware (Dienstag). Later, volatile oil was crystallized with circulating cool water. The crystals were collected and used. To obtain highly pure ZER, recrystalization was conducted by using hexane and the solution left standing to evaporate. At each step, thin layer chromatography was adopted to examine purification of ZER. The purity of the extracted ZER was >98%. For further pharmacological analyses, the crystals of ZER were kept.

### 2.2. STZ-Diabetic Rats

Male Wistar rats (8–10 weeks of age, 200–250 g) were obtained from the National Laboratory Animal Center (Tainan City, Taiwan). To induce diabetes, rats were given a single intravenous injection of 60 mg/kg streptozotocin (STZ; Sigma-Aldrich, Inc., St. Louis, MO, USA). Animals were considered to be diabetic if they had plasma glucose concentrations of 350 mg/dL or greater, along with polyuria and other diabetic features. After the injection of STZ, all studies were conducted for two weeks. All animal procedures were performed according to the Guidelines for the Care and Use of Laboratory Animals of the National Institutes of Health (United States), as well as the guidelines of the Animal Welfare Act. The study was conducted with the approval of the Institutional Animal Care and Use Committee (IACUC) at Tajen University (approval number: IACUC 103-13; approval date: 14 October 2014).

### 2.3. Treatment Protocols

The animals were randomly divided into four groups (*n* = 8 per group). In the treatment group, STZ-diabetic rats were dosed with 40 mg/kg ZER in distilled water (1.5 mL/kg) by oral gavage once daily for eight weeks. The dosage regime was based on a previous report that ZER at this dosage was potentially effective on improving diabetic nephropathy in STZ-diabetic rats [18]. As a positive control, a group of STZ-diabetic rats were treated orally for eight weeks with aminoguanidine (AG; purity ≥99.0%, Sigma-Aldrich, Inc.) at the daily dose of 50 mg/kg. As for the dose of AG, it was based on studies of long-term treatment for DR in Zucker diabetic fatty rats [23]. A vehicle-treated group of STZ-diabetic rats and normal rats were treated with 1.5 mL/kg distilled water only over the same treatment period. Throughout the entire treatment period, animals had free access to standard rat diet (Harlan Teklad, Madison, WI, USA; Cat. No. 2018) and water. 

At the end of the eight-week treatment, the rats were weighed, fasted overnight and anesthetized using an intraperitoneal injection of sodium pentobarbital (60 mg/kg). While under anesthesia, they were painlessly sacrificed and blood was collected from the abdominal aorta of each animal into heparin sample bottles. After rat eyes from each group were removed and washed with cold normal saline, they were used for the eye homogenate and histopathological examinations.

### 2.4. Blood Sampling and Analysis

The heparin anti-coagulated blood samples were centrifuged at 2000× *g* for 10 min at 4 °C, and later their plasma was collected and stored for subsequent analysis. With a diagnostic kit from BioSystem (Barcelona, Spain; Cat. No. COD12503), plasma glucose concentration was determined. Commercial enzyme-linked immunosorbent assay (ELISA) kits were used to quantify glycosylated hemoglobin (HbA_1c_) levels (Integrated Bio Ltd., Taipei, Taiwan; Cat. No. CSB-E08140r). All analyses were performed in accordance with the instructions provided by the manufacturers.

### 2.5. Retinal AGEs Determination

The rats’ eyes were collected and the leftones were used for the measurements of AGEs at the end of treatment. Retinas were lysed in ice-cold RIPA buffer (50 mmol/L, Tris-HCl (pH 8), 150 mmol/L NaCl, 1 mmol/L EDTA, 0.1% SDS, 1% IGEPAL and 0.5% sodium deoxicholate) containing protease inhibitor cocktails (Sigma-Aldrich, Inc.; Cat. No. P83490) and centrifuged for 15 min at 10,000× *g* at 4 °C. The supernatants were collected and assayed for protein content by Bio-Rad DC protein assay kit (Bio-Rad Laboratories, Milan, Italy). Rat AGEs ELISA kit (Cat. No. MBS261131) was obtained from MyBioSource, Inc. (San Diego, CA, USA). All of the above measurements were performed in triplicate. For protein concentration, tissue sample concentration was calculated from a standard curve and corrected (Abcam plc., Cambridge, UK).

### 2.6. Retinal Morphologic Study

Four micrometer sections of eyes on glass slides were deparaffinized in xylene and serially treated with 100%, 96%, and 70% ethanol. Hematoxylin and eosin (H & E) stained slides for light microscopy. Retinal thickness was measured as the length (μm) from outer limiting membrane to inner limiting membrane, 1 mm away from the optic nerve on the superior side [24]. With an attached digital camera (C3040-AD6, Olympus, Tokyo, Japan), all measurements were performed under a light microscope.

### 2.7. Protein Extraction and Western Blot Analyses

Retinas were homogenized in 1 mL of ice-cold hypotonic buffer A (10 mmol/L of HEPES, 10 mmol/L of KCl, 2 mmol/L of MgCl_2_, 1 mmol/L of DTT, 0.1 mmol/L of EDTA, and 0.1 mmol/L of phenylmethylsulfonylfluoride; pH 7.8). A solution of 80 μL of 10% Nonidet P-40 was added to the homogenates, and the mixture was centrifuged for 2 min at 14,000× *g* at 4 °C. Before immunoblotting, the protein concentration of each tissue was determined using a Bio-Rad protein assay kit (Bio-Rad Laboratories, Tokyo, Japan) and bovine serum albumin was used as a standard to ensure equal loading among lanes. The tissue lysates containing 40–50 mg protein were electrophoresed through 8%, 12%, and 15% sodium dodecyl sulfate-polyacrylamide gels. According to the manufacturer’s instructions, separated proteins were electrophoretically transferred to a nitrocellulose membrane, blocked with 5% skim milk solution for 1 hour, and incubated with primary antibodies to RAGE (Cat. No. sc-5563), TNF-α (Cat. No. sc-1348), IL-1β (Cat. No. sc-7884), IL-6 (Cat. No. sc-1266), VEGF (Cat. No. sc-1836), ICAM-1 (Cat. No. sc-8439), VCAM-1 (Cat. No. sc-8304), and glyceraldehyde-3-phosphate dehydrogenase (GAPDH, Cat. No. sc-20357) at 4 °C overnight, respectively. All antibodies were purchased from Santa Cruz Biotechnology, Inc. (Santa Cruz, CA, USA) and used at a dilution of 1:1000. After three 5-min washes in TBST (20 mmol/L Tris-HCl, pH 7.5, 150 mmol/L NaCl, and 0.05% Tween 20), membranes were incubated with the appropriate peroxidase-conjugated secondary antibodies. With the ECL Advance Western Blotting detection kit (Cat. No. RPN2135; GE Healthcare Life Sciences, Pittsburgh, PA, USA), the membranes were washed three times in TBST and visualized on X-ray film. Band densities were determined using ATTO Densitograph Software (ATTO Corporation, Tokyo, Japan) and quantified as the ratio to GAPDH. The mean value for samples was adjusted to a value of 1.0 from the vehicle-treated normal rats on each immunoblot, expressed in densitometry units. Then, all experimental sample values were expressed to this adjusted mean value. From 4 independent experiments, tissue sections were sampled.

### 2.8. Real-Time Polymerase Chain Reaction (PCR)

Under the manufacturer’s protocol, total RNA was extracted from rat retinas using a Trizol reagent (Invitrogen; Boston, MA, USA). RNA, whose integrity was verified by agarose gel electrophoresis using ethidium bromide for visualization, was quantified by A260. For the reverse transcriptase reaction, 1 μg of total RNA per sample and 8.5 μg/μL random hexamer primers were heated at 65 °C for 5 min and then quenched on ice. This mixture was combined with 500 μmol/L each of dATP, dTTP, dCTP, and dGTP, 10 mmol/L DTT, 20 mmol/L Tris-HCl (pH 8.4), 50 mmol/L KCl, 5 mmol/L MgCl_2_, 40 units of RNaseOUT™ recombinant ribonuclease inhibitor (Invitrogen) and 100 units SuperScript III reverse transcriptase (Invitrogen). Samples were subjected to DNase (Promega; Madison, WI, USA) treatment at 37 °C for 20 min in a GeneAmp 9700 Thermal Cycler (Applied Biosystems; Foster City, CA, USA) and then held at 4 °C. Next, aliquots were taken for instant use in PCR. The remainder of the cDNA was stored at −20 °C. In a fluorescent temperature Lightcycler 480 (Roche Diagnostics; Mannheim, Germany), mRNA expression was measured by quantitative real-time PCR. Primer sequences were as follows: 5′-AAGCCCCTGGTGCCTAATGAG-3′ (forward) and 5′-CACCAATTGGACCTCCT-CCA-3′ (reverse) for RAGE; 5′-ACACCATGAGCACGGAAAGC-3′ (forward) and 5′-CCGCCACGAGCAGGAA-3′ (reverse) for TNF-𝛼; 5′-AATGGACAGAACATAAGCCAACA-3′ (forward) and 5′-CCCAAGGCCACAGGGAT-3′ (reverse) for IL-1β; 5′-GTTGCCTTCTTGGGACTGATG-3′ (forward) and 5′-ATACTGGTCTGTTGTGGGTGGT-3′ (reverse) for IL-6; 5′-ACAGGG AAG ACA ATG GGATGA-3′ (forward) and 5′-GGGCCAGGGATGGGT TT-3′ (reverse) for VEGF; 5′-CGGGTTTGGGCTTCTCC-3′ (forward) and 5′-GCCACTGCTCGTCCACATAG-3′ (reverse) for ICAM-1; 5′-ATCTTCGGAGCCTCAACGG-3′ (forward) and 5′-CCAATCTGAGCGAGCGTTT-3′ (reverse) for VCAM-1; 5′-TGTGATGGTGGGAATGGGTCAG-3′ (forward) and 5′-TTTGATGTCACGCACGATTTCC-3′ (reverse) for β-actin. Primers were designed using Primer Express Software version 2.0 System (Applied Biosystems; Foster City, CA, USA). The PCR reaction was conducted under the following cycling protocol: 95 °C for 5 min, accompanied by 45 cycles of 95 °C for 5 s, 58 °C for 15 s, and 72 °C for 20 s. To identify the specific PCR products, dissociation curves were run after amplification. Based on the delta-delta Ct method, the mRNA expression levels were normalized to β-actin mRNA levels and calculated [25].

### 2.9. Quantification of NF-κB Activation

Nuclear extracts of retina of the above-mentioned groups were prepared with the aid of the nuclear extract kit (Active Motif, Carlsbad, CA, USA). NF-κB activity was determined, TransAM^®^ NF-κB p65 transcription factor assay kit (Active Motif) implemented under the procedures provided by the manufacturer. Twenty micrograms of retinal nuclear extracts were incubated with an oligonucleotide containing the NF-κB consensus site, and later with monoclonal and secondary antibody directed against the NF-κB p65 subunit. Reaction was quantified at 450 nm.

### 2.10. Quantification of Apoptosis

Cell death detection ELISA kit (Cat. No. 1544675; Roche, Germany) was used to quantitatively detect the cytosolic histone-associated DNA fragmentation, based on the manufacturer’s instructions. Retinal cytoplasmic extracts (25 μL) were used as an antigen source in a sandwich ELISA. The change in color wasmeasured at a wavelength of 405 nm by using a Dynex MRX plate reader controlled through PC software (Revelation; Dynatech Laboratories, Chantilly, VA, USA). The optical density (OD) reading was then normalized to the total amount of protein in the sample and the data were reported as an apoptotic index (OD_405_/mg protein) to indicate the level of cell death.

### 2.11. Statistical Analysis

Data are expressed as the mean ± standard error mean (SEM). Statistical analysis was performed with one-way analysis of variance. Dunnett range post-hoc comparisons were used to determine the source of significant differences, where appropriate. All statistical analyses were performed using SPSS for Windows (version 21.0; IBM Corporation, Armonk, NY, USA). A *p*-value < 0.05 was considered statistically significant.

## 3. Results

### 3.1. Changes in the Body Weight, Plasma Levels of Glucose and Glycosylated Hemoglobin

After the 8-week treatment, compared with normal rats whose blood glucose levels were significantly higher, the weight gain in STZ-diabetic rats was significantly less (Table 1). During the experimental period, the reduction of body weight in STZ-diabetic rats receiving ZER or AG was not obvious. When STZ-diabetic rats were treated with ZER (32.5% ± 5.7%) for 8 weeks, it was obvious that the blood glucose lowered effect (Table 1). 

In STZ-diabetic rats, the value of HbA_1c_ was markedly higher compared with normal rats (Table 1). Treatment ZER for 8 weeks decreased the levels of HbA_1c_ in STZ-diabetic rats by 29.2% ± 3.4% relative to the value in STZ-diabetic rats that received vehicle (Table 1). 

It was found that the plasma levels of glucose and HbA_1c_ in STZ-diabetic rats treated with AG were similar to the values of vehicle-treated diabetic animals (Table 1).

### 3.2. Retinal Morphology

**Figure 1:**
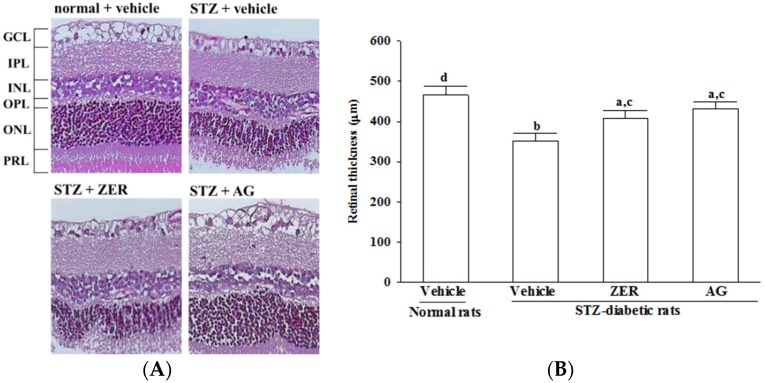
Presentation of (**A**) retinal morphology and (**B**) thickness in rats. STZ-diabetic rats were administered 40 mg/kg/day ZER (STZ + ZER), or 50 mg/kg AG (STZ + AG) by oral gavage once daily for three months. Another group of STZ-diabetic rats (STZ + vehicle) and normal rats (normal + vehicle) were administered the same volume of vehicle used to prepare the test medication solutions. All the representative images of hematoxylin and eosin (H & E) stained retinas were taken at ×200 magnification. GCL; ganglion cell layer; INL, inner nuclear layer; IPL, inner plexiform layer; NFL, nerve fiber layer; OPL, outer plexiform layer; ONL, outer nuclear layer; PRL, photoreceptor layer. Values (mean ± SEM) were obtained from eight animals in each group. ^a^ *p* < 0.05 and ^b^ *p* < 0.01 compared to vehicle-treated normal rats, respectively; ^c^ *p* < 0.05 and ^d^ *p* < 0.01 compared to the values of vehicle-treated STZ-diabetic rats, respectively.

The morphological changes in retinas in rats are shown in Figure 1A. Retina of normal rats exhibited normal arrangement of retinal cell layers including the layers of nerve fiber, ganglion cell, and nuclear layer. Both layers of nerve fibers and ganglion cells attained considerable atrophy in retina of STZ-dibetic rats (Figure 1A). Administration STZ-diabetic rats with ZER or AG ameliorated the dramatic histological alterations but did not reach the normal structural pattern (Figure 1A). Furthermore, the retinas of STZ-diabetic rats were significantly thinner than those of normal rats; ZER or AG treatment attenuated this impairment (Figure 1B).

### 3.3. The Gene Expression of Retinal AGEs and RAGE

In observing in the vehicle-treated counterparts, the retinal protein level of AGEs had significantly increased in the STZ-diabetic rats compared with that in normal rats, which were downregulated by ZER treatment, with a decrease of 41.5% ± 4.3% (Table 1). AG treatment also decreased retinal AGEs protein level to 49.9% ± 5.6% of those in vehicle-treated STZ-diabetic rats (Table 1).

**Figure 2:**
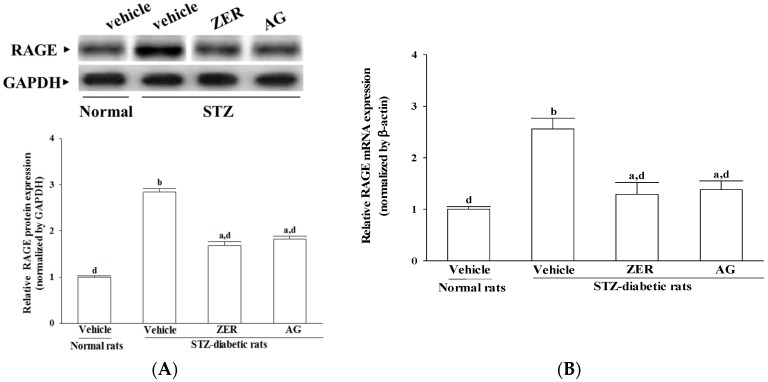
Effects of the treatments on the (**A**) protein and (**B**) mRNA levels of receptors for advanced glycosylated end products (RAGE) in retina of rats. STZ-diabetic rats were administered 40 mg/kg/day ZER, or 50 mg/kg AG by oral gavage once daily for three months. Another group of STZ-diabetic rats and normal rats were administered the same volume of vehicle used to prepare the test medication solutions. Data are mean ± SEM from eight rats per group, and the experiments were repeated independently at least three times with similar results. ^a^ *p* < 0.05 and ^b^ *p* < 0.01 compared to vehicle-treated normal rats, respectively; ^c^ *p* < 0.05 and ^d^ *p* < 0.01 compared to the values of vehicle-treated STZ-diabetic rats, respectively.

Retinal protein and mRNA levels of RAGE in STZ-diabetic rats were clearly higher than those of the normal rats and were downregulated by ZER (40 mg/kg/day) treatment (40.8% ± 5.3% and 49.6% ± 6.1% decreases, respectively; Figure 2). The protein and mRNA levels of retinal RAGE in STZ-diabetic rats receiving AG treatment were reduced by 35.9% ± 4.7% and 46.1% ± 5.2%, respectively, as compared with their vehicle-treated counterparts (Figure 2).

### 3.4. The Gene Expression of Retinal Inflammatory Cytokines and Chemokines

**Figure 3:**
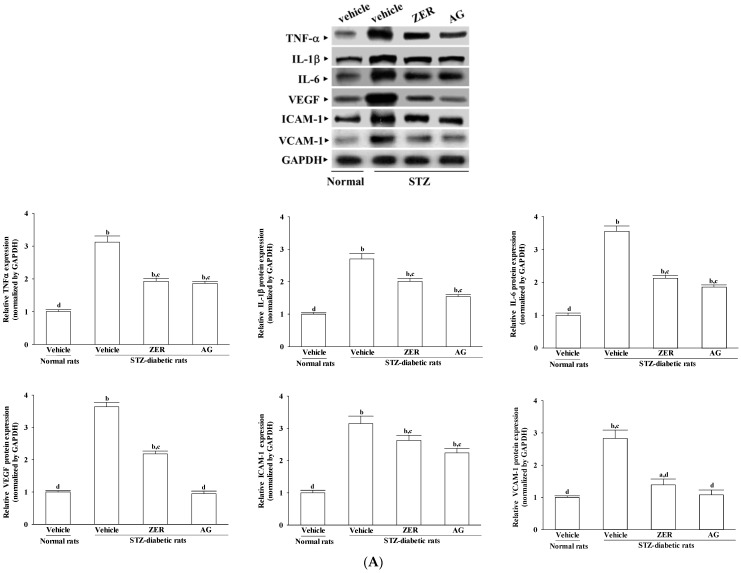

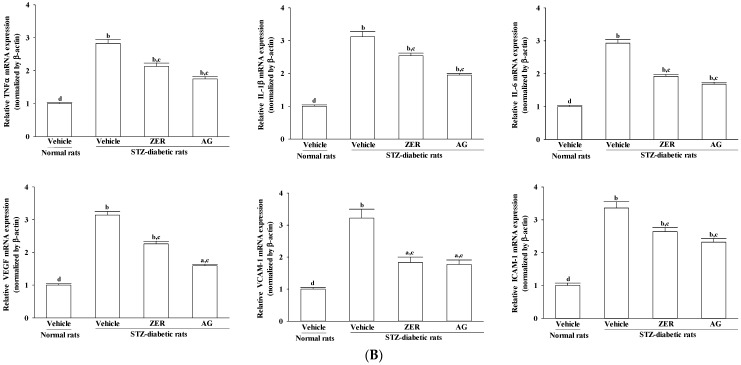
Effects of the treatments on the (**A**) protein and (**B**) mRNA levels of inflammatory cytokines and chemokines in retina of rats. STZ-diabetic rats were administered 40 mg/kg/day ZER, or 50 mg/kg AG by oral gavage once daily for three months. Another group of STZ-diabetic rats and normal rats were administered the same volume of vehicle used to prepare the test medication solutions. Data are mean ± SEM from eight rats per group, and the experiments were repeated independently at least three times with similar results. ^a^ *p* < 0.05 and ^b^ *p* < 0.01 compared to vehicle-treated normal rats, respectively; ^c^ *p* < 0.05 and ^d^ *p* < 0.01 compared to the values of vehicle-treated STZ-diabetic rats, respectively.

STZ-diabetic rats had higher retinal protein and mRNA levels of TNF-α, IL-1β, and IL-6 as compared to those of normal rats (Figure 3). Both the protein and mRNA levels of retinal TNF-α, IL-1β, and IL-6 in ZER (40 mg/kg/day)-treated STZ-diabetic rats were lower than those of their vehicle-treated counterparts (Figure 3). Compared to those of their vehicle-treated counterparts, AG treatment reduced retinal protein and mRNA levels of TNF-α, IL-1β, and IL-6 in STZ-diabetic rats as well (Figure 3).

Relative to the levels in the normal rats, the retinal protein and mRNA levels of VEGF in STZ-diabetic rats respectively were significantly higher to 3.2 and 3.6 fold. After daily treatment with ZER (40 mg/kg/day), retinal protein and mRNA levels of VEGF were reduced by 40.1% ± 5.4% and 28.2% ± 3.9% relative to the levels in their vehicle-treated counterparts, respectively (Figure 3). Likewise, treatment of STZ-diabetic rats with AG dramatically reduced retinal VEGF level (Figure 3).

Compared to the normal group, STZ-diabetic rats had more retinal protein and higher mRNA levels of ICAM-1 and VCAM-1 (Figure 3A,B). STZ-diabetic rats receiving treatment with ZER (40 mg/kg/day), compared with those in vehicle-treated counterparts, respectively resulted in a 16.5% ± 3.2% and 50.9% ± 4.6% reduction of retinal ICAM-1 and VCAM-1 protein expression (Figure 3A). Relative to the expression levels in vehicle-treated counterparts, the retinal mRNA levels of ICAM-1 and VCAM-1 in STZ-diabetic rats which had received ZER (40 mg/kg/day) treatment respectively decreased to 21.4% ± 3.7% and 43.2% ± 4.9% (Figure 3B). It showed similar results for AG-treated STZ-diabetic rats (Figure 3A,B).

### 3.5. NF-kB Activity and Apoptosis Rate

**Figure 4:**
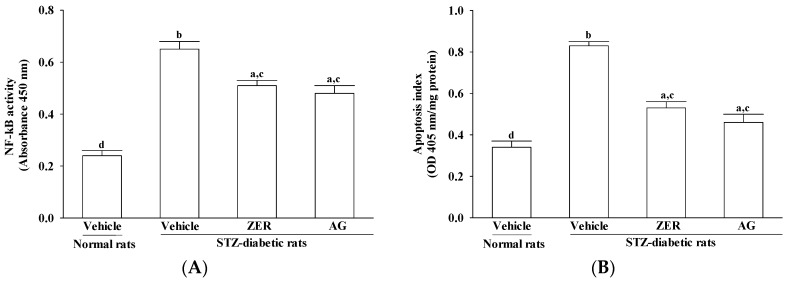
Effects of the treatments on the retinal NF-κB activity (**A**) and apoptosis rate (**B**) in rats. STZ-diabetic rats were administered 40 mg/kg/day ZER, or 50 mg/kg AG by oral gavage once daily for three months. Another group of STZ-diabetic rats and normal rats were administered the same volume of vehicle used to prepare the test medication solutions. Data are mean ± SEM from eight rats per group, and the experiments were repeated independently at least three times with similar results. ^a^ *p* < 0.05 and ^b^ *p* < 0.01 compared to vehicle-treated normal rats, respectively; ^c^ *p* < 0.05 and ^d^ *p* < 0.01 compared to the values of vehicle-treated STZ-diabetic rats, respectively.

In comparing to the STZ-induced upregulation in STZ-diabetic rats after 8 weeks of treatment with 40 mg/kg/day ZER, that of NF-κB activity reduced 21.5% ± 5.3% (Figure 4A). The higher NF-κB activity reversed in the retina after 8 weeks of treatment with AG, which decreased 46.1% ± 4.9% compared to those in vehicle-treated STZ-diabetic rats (Figure 4A). 

STZ caused a 2.4-fold increase of retinal apoptosis rate and AG treatment attenuated this enhancements by 44.5% ± 6.2% when compared to the level seen in the normal group (Figure 4B). In fact, the apoptosis rate significantly decreased to 63.8% ± 5.8% in the retinas of STZ-diabetic rats with ZER (40 mg/kg/day) treatment relative to the levels in vehicle-treated counterparts (Figure 4B). 

## 4. Discussion

It has been reported that a decrease in retinal thickness could arise from retinal damage and the progression of DR might result from structural disarrangement in diabetes, which contributes to vision loss [26]. The results of retinal morphologic study showed that the total retinal thickness and the combined nerve fiber layers and ganglion cell layer were maintained in the ZER-treated STZ-diabetic rats compared with the untreated diabetic rats. Prevention from structural abnormality of the retina in diabetic rats may be a validation to support the beneficial potential of ZER in amelioration of DR.

Effective blood glucose control is the key to preventing or reversing diabetic complications and improving quality of life in diabetic patients. ZER lowered the levels of blood glucose successfully and, meanwhile, the levels of HbA1c in STZ-diabetic rats [18]. The concentration of HbA1c is considered to be a good marker for diagnosis and prognosis of diabetes complications [27]. Thus, the antidiabetic properties of ZER observed make it candidate of a therapeutic supplement that can reduce diabetic-related complications. In conclusion, the evaluation of ZER on the modulation of the mechanism involved in DR is needed.

Glucose participates in nonenzymatic glycation of proteins to produce AGEs [28]. It has been already reported that both AGEs and RAGE have been localized to the retinal vasculature and vascular endothelial cells [29]. Furthermore, it has been demonstrated that AGEs contribute to altering protein function, interfering with the extracellular matrix function, and elaboration of cytokines. In addition, it was reported that AGEs are directly associated with the apoptotic cell death of retinal neuronal cells [30]. Experiments on diabetic animals also showed that AG, a selective inhibitor of AGEs, prevented acellular capillaries, the retinal microaneurysms, and pericyte loss in the diabetic dogs that further proved the role of AGEs in the pathogensis of DR [26]. In fact, 8 weeks’ of ZER treatment alleviated not only the overexpression of AGEs but also the higher levels of RAGEs in retinas of STZ-diabetic rats, and the effect was comparable to that of AG. These results suggest that ZER treatment results in the decrease of interaction between AGEs and RAGE and a decline in cellular damage mediated by AGEs. It seems that ZER exhibited the properties of an AGE inhibitor in retinal tissue.

The theory that an inflammatory reaction is involved in the pathological process of DR has been recognized by scholars. Proinflammatory cytokines (such as TNF-α and interleukins) activate endothelium to increase expression of adhesion molecules (such as ICAM-1, VCAM-1) and chemokines, by which leukocytes/monocytes were mediated to attach to the vessel wall and transmigrate through the endothelium [7]. VEGF, a angiogenic cytokine, is known to be a key molecule leading to retinal permeability and breakdown of blood–retinal barrier in diabetes and other retinal diseases [31]. In addition, VEGF has been shown to promote endothelial cell expression of ICAM-1, leading to leukocyte activation and cytokine release, thereby causing further increases in VEGF expression and amplification of the inflammatory response [31]. Special attention is focused on pharmacological agents with anti-inflammatory effect concerning the involvement of the low-grade chronic inflammatory processes in the pathogenesis of DR, [9]. On the conditions of our experiment, we confirmed that ZER suppressed the gene expression of a series of proinflammatory cytokines and chemokines which may consequently result in decreased adhesion molecules expression in retina of STZ-diabetic rats. The higher VEGF expression in retinae of STZ-diabetic rats were also decreased in rats receiving ZER treatment. Therefore, the effect of ZER to attenuate inflammation may be involved in a possible mechanism for preventing the progression of DR, by reducing the release of inflammatory mediators and/or inhibiting the expression of adhesion molecules in the DR. Moreover, ZER has offered a protective effect on diabetes induced vasculopathy via the anti-angiogenic factor.

AGEs have been reported to interact with RAGE, inducing the subsequent activation of NF‑κB [31]. NF-κB has been considered a widely expressed inducible transcriptional factor which is an important regulator of many genes involved in mammalian inflammatory and immune responses, including proliferation and apoptosis [32]. Diabetes has been shown to activate NF-kB in rodent retinas, and to cause migration of the p65 subunit into nuclei of retinal endothelial cells, pericytes, ganglion cells, or cells of the inner nuclear layer [33,34,35]. The inhibition of NF-κB might be a critical step for the prevention of AGE-RAGE-mediated apoptosis and inflammation associated gene expressions in DR. Our observations demonstrated that ZER treatment almost completely inhibited NF-κB activation in retinas of STZ-diabetic rats. Another verification to support the beneficial effect of ZER in preventing diabetes complications is the reduction of retinal apoptosis rates after 8-weeks’ ZER administration. These results suggest that the inhibition of ZER on retinal inflammation in diabetic rats may be associated with suppression of NF-κB activation and that it eventually decreased tissues apoptosis rates. Therefore, it was suggested that the blockade of the AGE-RAGE axis and suppression of NF-κB activation may be the targets of ZER for DR.

**Figure 5:**
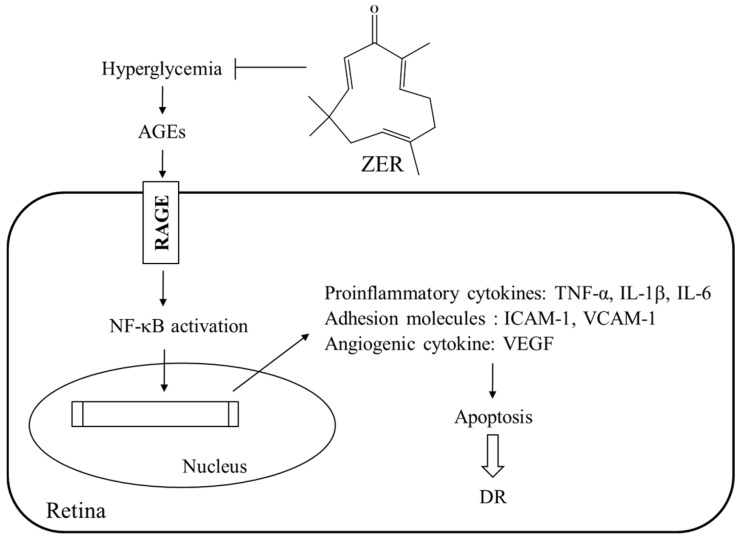
The possible action mechanisms of ZER on the amelioration of streptozotocin-induced retinal damage in rats. ZER showed the retinalprotective effects via reducing hyperglycemia to inhibit AGEs and RAGE axis, and thereby resulting in downregulation the expressions of proinflammatory cytokines, adhesion molecules and angiogenic cytokine, that in turn retards apoptosis, and consequently lessens damage in diabetic retinal tissue.

The findings of the present study are of merit in revealing that ZER can ameliorate or retard the development of DR. There have been a large number of therapies that have been found to essentially totally inhibit retinal vascular disease including capillary degeneration or neural disease in diabetes even in the presence of continued hyperglycemia [36]. A possible mechanism of action of ZER’s amelioration of hyperglycemia-induced retinal damage in STZ-diabetic rats is indicated in Figure 5. It seems that ZER has an antidiabetic property with plasma glucose-lowering action to reduce hyperglycemia-induced AGE-RAGE pathway, and resulting in downregulation of NF-κB-mediated inflammatory signals, and consequently attenuation of apoptosis in diabetic retinal tissue. Therefore, we suggest that ZER has protective effects on several pharmacological targets in DR. The effect of ZER mainly responsible for benefit effect on DR are to be identified in the future research work.

Medicinal plants produced several useful biological activities, few or none of which is known concerning their toxicities in humans. As such, the inclusion of toxicological evaluation at preclinical stage will assure its safe usage in humans as a medicine [37]. The median lethal dose value (1.84 g/kg) obtained in rats was a clear indication that ZER could be safe for use in one dose treatment [38]. Further studies are needed to clarify ZER toxicity to rat at the effective dosage used for treating DR. Nevertheless, this study provides an important pharmacological basis for the amelioration of the microvascular complications associated with diabetes.

## 5. Conclusions

The present study has shown that after regular consumption in the specified dosage, ZER has potential in amelioration of hyperglycemia-induced retinal damage in a rat model of DR. The antiapoptosis and anti-inflammatory properties of ZER could attribute to its protective effects, the inhibitory effect on AGEs/RAGE signaling, and NF-κB activation in retina, with additional antihyperglycemia effect.

## Figures and Tables

**Table 1:** Changes in the body weight, plasma levels of glucose and glycosylated hemoglobin (HbA1C) and retinal advanced glycosylated end products (AGEs) content in experimental animals at the end of the 8-week treatment.

Groups	Body Weight (g/rat)	Plasma Glucose (mg/dL)	HbAlc (%)	retinal AGEs (µg/g)
Normal rats				
vehicle-treated	323.8 ± 13.6 ^d^	93.7± 6.1 ^d^	4.9 ± 0.5 ^d^	9.8 ± 0.8 ^d^
STZ-diabetic rats				
vehicle-treated	212.6 ± 13.9 ^b^	414.5 ± 12.3 ^b^	16.1 ± 1.3 ^b^	31.2 ± 2.8 ^b^
ZER-treated	280.2 ± 14.3 ^a,c^	279.6 ± 11.7 ^b,d^	11.4 ± 0.9 ^b,c^	18.3 ± 3.1 ^a,c^
AG-treated	275.3 ± 13.7 ^a,c^	393.2 ± 13.1 ^b^	15.3 ± 1.1 ^b^	15.6 ± 2.9 ^a,c^

Streptozotocin (STZ)-diabetic rats were dosed by oral gavage once per day for 8 weeks with 40 mg/kg/day zerumbone (ZER), or 50 mg/kg aminoguanidine (AG). Normal or STZ-diabetic rats receiving vehicle treatment were given the same volume of vehicle (distilled water) used to prepare the test medication solutions. Values (mean ± standard error mean (SEM)) were obtained for each group of eight animals. ^a^ *p* < 0.05 and ^b^ *p* < 0.01 compared to the values of vehicle-treated normal rats, respectively; ^c^ *p* < 0.05 and ^d^ *p* < 0.01 compared to the values of vehicle-treated STZ-diabetic rats, respectively.
